# Intrinsic braking role of descending locus coeruleus noradrenergic neurons in acute and chronic itch in mice

**DOI:** 10.1186/s13041-020-00688-0

**Published:** 2020-10-27

**Authors:** Keisuke Koga, Yuto Shiraishi, Ryo Yamagata, Hidetoshi Tozaki-Saitoh, Miho Shiratori-Hayashi, Makoto Tsuda

**Affiliations:** 1 Department of Life Innovation, Graduate School of Pharmaceutical Sciences, Kyushu University, Fukuoka, 812-8582 Japan; 2 Department of Neurophysiology, Hyogo College of Medicine, Nishinomiya, Hyogo 663-8501 Japan; 3 Department of Molecular and System Pharmacology, Graduate School of Pharmaceutical Sciences, Kyushu University, 3-1-1 Maidashi, Higashi-ku, Fukuoka, 812-8582 Japan

**Keywords:** Descending noradrenaline neurons, Locus coeruleus, Spinal dorsal horn, Gastrin-releasing peptide receptor-expressing neurons, Itch, Mouse

## Abstract

Itch is defined as an unpleasant sensation that provokes a desire to scratch. Our understanding of neuronal circuits for itch information transmission and processing in the spinal dorsal horn (SDH) has progressively advanced following the identification of SDH neuron subsets that are crucial for scratching behavior in models of itch. However, little is known about the control of acute and chronic itch by descending signals from the brain to the SDH. In this study, using genetic approaches that enable cell-type and circuit-specific functional manipulation, we reveal an intrinsic potential of locus coeruleus (LC)-noradrenergic (NAergic) neurons that project to the SDH to control acute and chronic itch. Activation and silencing of SDH-projecting LC-NAergic neurons reduced and enhanced scratching behavior, respectively, in models of histamine-dependent and -independent acute itch. Furthermore, in a model of chronic itch associated with contact dermatitis, repetitive scratching behavior was suppressed by the activation of the descending LC-NAergic pathway and by knocking out NA transporters specific to descending LC-NAergic neurons using a CRISPR-Cas9 system. Moreover, patch-clamp recording using spinal slices showed that noradrenaline facilitated inhibitory synaptic inputs onto gastrin-releasing peptide receptor-expressing SDH neurons, a neuronal subset known to be essential for itch transmission. Our findings suggest that descending LC-NAergic signaling intrinsically controls acute and chronic itch and provide potential therapeutic strategies for the treatment of acute and chronic itch.

## Introduction

Itch is defined as an unpleasant cutaneous sensation that provokes the desire to scratch, and scratching can transiently relieve such itching sensations [1]. However, in pathological conditions such as atopic and contact dermatitis, itch sensation becomes intense and chronic, which leads to excessive, repetitive scratching. As the existing treatments (e.g., antihistamines) are largely ineffective, the elucidation of the mechanisms underlying chronic itch and the development of novel therapeutic agents are crucial.

Pruriceptive information is conveyed via primary afferents from the skin and processed in the spinal dorsal horn (SDH). Recent studies have progressively advanced our understanding of the mechanism underlying neuronal circuits for itch transmission in the nervous system [2–5]. Specifically, gastrin-releasing peptide receptor (GRPR)-expressing (GRPR^+^) neurons in the SDH act as a hub for converging pruriceptive information and are essential for producing scratching behaviors in diverse models of acute and chronic itch [6–8]. Furthermore, similar to the regulation of nociceptive transmission, pruriceptive transmission in the SDH has been considered to also be remotely controlled by the brain through descending neuronal pathways. Noradrenaline (NA) and serotonin (5-HT) are the two major monoamines that are utilized as neurotransmitters in the descending pathways. A recent study has shown that scratching behavior is suppressed in mice with decreased spinal 5-HTergic terminals and lacking the enzyme for 5-HT synthesis [9]. Activation of 5-HT1A receptors potentiates the excitation of GRPR^+^ neurons via the enhancement of gastrin-releasing peptide (GRP)-induced responses. This suggests that the activation of descending 5-HTergic pathways facilitates itch transmission via GRPR signaling in the SDH [9]. On the other hand, intrathecal injection of agonists for α_1_- or α_2_-adrenaline receptors (ARs) has been shown to inhibit acute itch-related scratching behavior [10]. It has also been reported that antidepressants that can increase spinal NA and/or 5-HT levels reduce scratching in mouse models of chronic itch [11] and in humans [12, 13], suggesting that endogenous spinal NA plays an inhibitory role in itching. Supporting this, there is an inverse correlation between itch-related scratching behavior and spinal NA content [10]. The locus coeruleus (LC) is the major brain region that contains SDH-projecting NAergic cell bodies [14, 15]. While intrathecal treatment with 6-hydroxydopamine (6-OHDA), a neurotoxin that can cause the degeneration of catecholaminergic neurons, has been reported to exacerbate scratching behavior [10], there is no direct evidence for the role of SDH-projecting LC-NAergic neurons in controlling acute and chronic itch.

In this study, using genetic tools that enable cell-type and circuit-specific functional manipulation, we demonstrated for the first time the potential ability of SDH-projecting LC-NAergic neurons to regulate scratching behavior in models of acute and chronic itch and also to enable the development of drugs to relieve itch sensation.

## Results

### Chemogenetic manipulation of descending LC-NAergic neurons

**Fig. 1:**
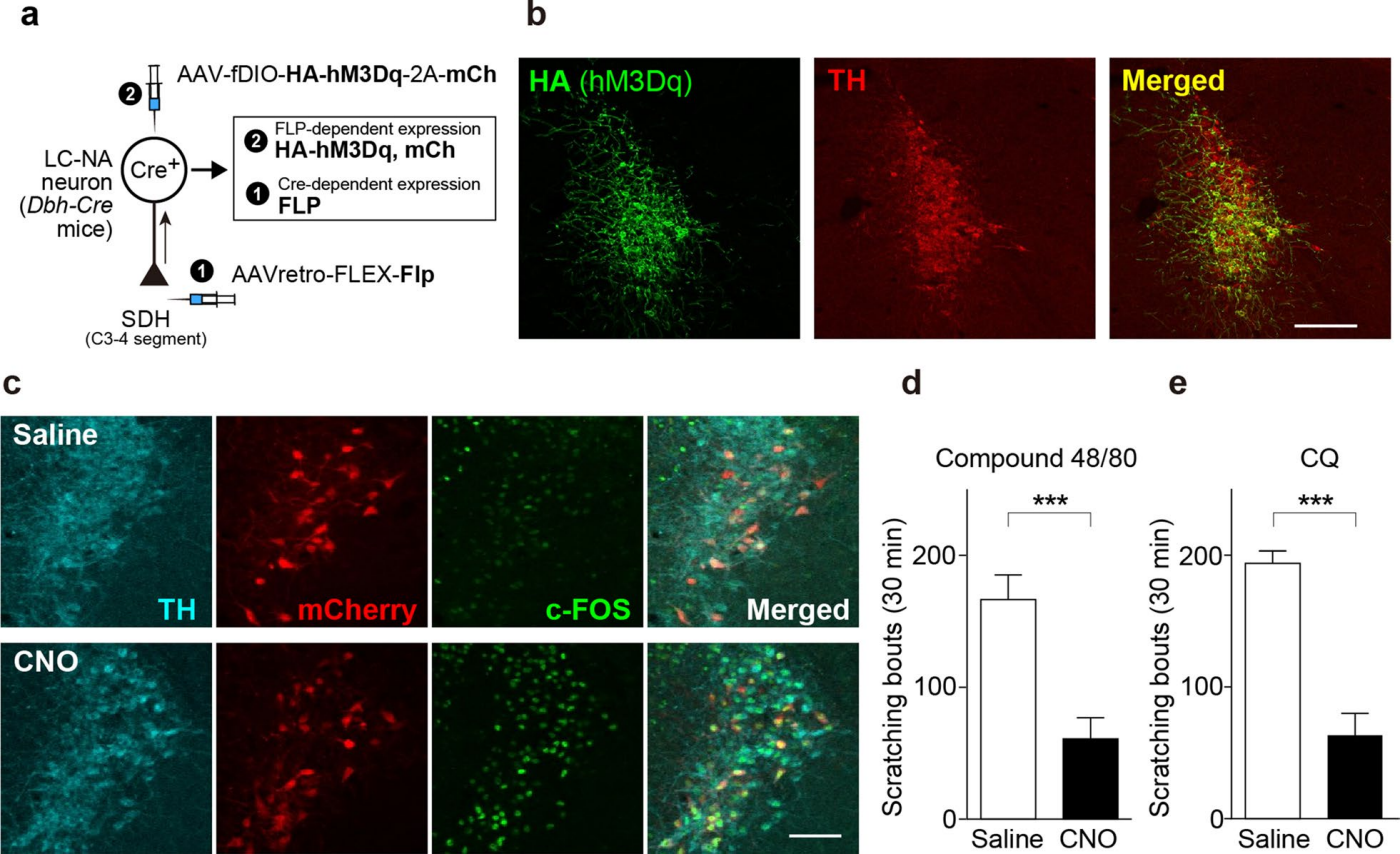
Chemogenetic activation of descending LC-NAergic neurons inhibits acute itch. **a.** A schematic illustration of the retrograde transduction strategy for hM3Dq expression in descending LC-NAergic neurons. **b** Representative images of hM3Dq (detected by HA immunostaining, green) and TH (red) expression in LC-NAergic neurons (visualized by TH immunostaining) in mice injected with the two AAV vectors. Scale bar, 200 μm. **c** Expression of c-FOS (green) in hM3Dq-expressing descending LC-NAergic neurons (visualized by mCherry and TH immunostaining) 2 h after administration of saline or CNO (10 mg/kg, i.p.) to AAV-injected mice. Scale bar, 100 μm. Scratching events induced by intradermal injection of compound 48/80 (50 μg/50 μl, **d**) or CQ (200 μg/50 μl, **e**) 20 min after CNO (10 mg/kg) or saline administration to AAV-injected mice (**d**: saline, n = 7; CNO, n = 8, **e**: saline, n = 5; CNO, n = 7, ***P < 0.001). Data show the mean ± SEM

To investigate the role of descending LC-NAergic neurons, we manipulated the activity of these neurons using chemogenetics [16]. First, we examined the effect of activation of LC-NAergic neurons on acute itch behavior. To express modified human muscarinic Gq-protein-coupled receptors (hM3Dq) [17] specifically in SDH-projecting LC-NAergic neurons, a retrograde adeno-associated viral (AAV) vector designed to express the recombinase flippase (FLP) in a Cre-dependent manner (AAVretro-FLEX-Flp) [18] was microinjected into the bilateral cervical SDH of *Dbh-Cre* mice [in which Cre is expressed NAergic neurons under the control of the promoter of dopamine-β-hydroxylase (DBH: an enzyme for NA biosynthesis)] [19]. Subsequently, an AAV vector designed to express hM3Dq in an FLP-dependent manner (AAV-fDIO-HA-hM3Dq-2A-mCherry) was injected into the bilateral LC (Fig. 1a). In these mice, hM3Dq (detected by HA or mCherry) were expressed mainly in the ventral LC (Fig. 1b), and 93.9 ± 1.1% of mCherry was colocalized with tyrosine hydroxylase (TH; a marker of NAergic neurons) (total: 424 neurons, n = 4 mice) (Fig. 1c). Intraperitoneal (i.p.) administration of the hM3Dq agonist, clozapine-N-oxide (CNO), in these mice induced c-FOS expression in mCherry^+^ neurons (Fig. 1c), confirming that the hM3Dq which was expressed in the LC-NAergic neurons is functional. Scratching behavior was then tested using models of histamine-dependent and -independent acute itch behavior caused by a single intradermal injection of compound 48/80 or chloroquine (CQ), respectively [6]. The basal spontaneous scratching for 15 min after CNO administration were not changed (saline, 4.0 ± 0.9, n = 12; CNO, 2.6 ± 0.9, n = 14; P = 0.304, unpaired t test), but CNO-administered mice showed a significant reduction of scratching responses induced by both compound 48/80 (Fig. 1d) and CQ (Fig. 1e).

**Fig. 2:**
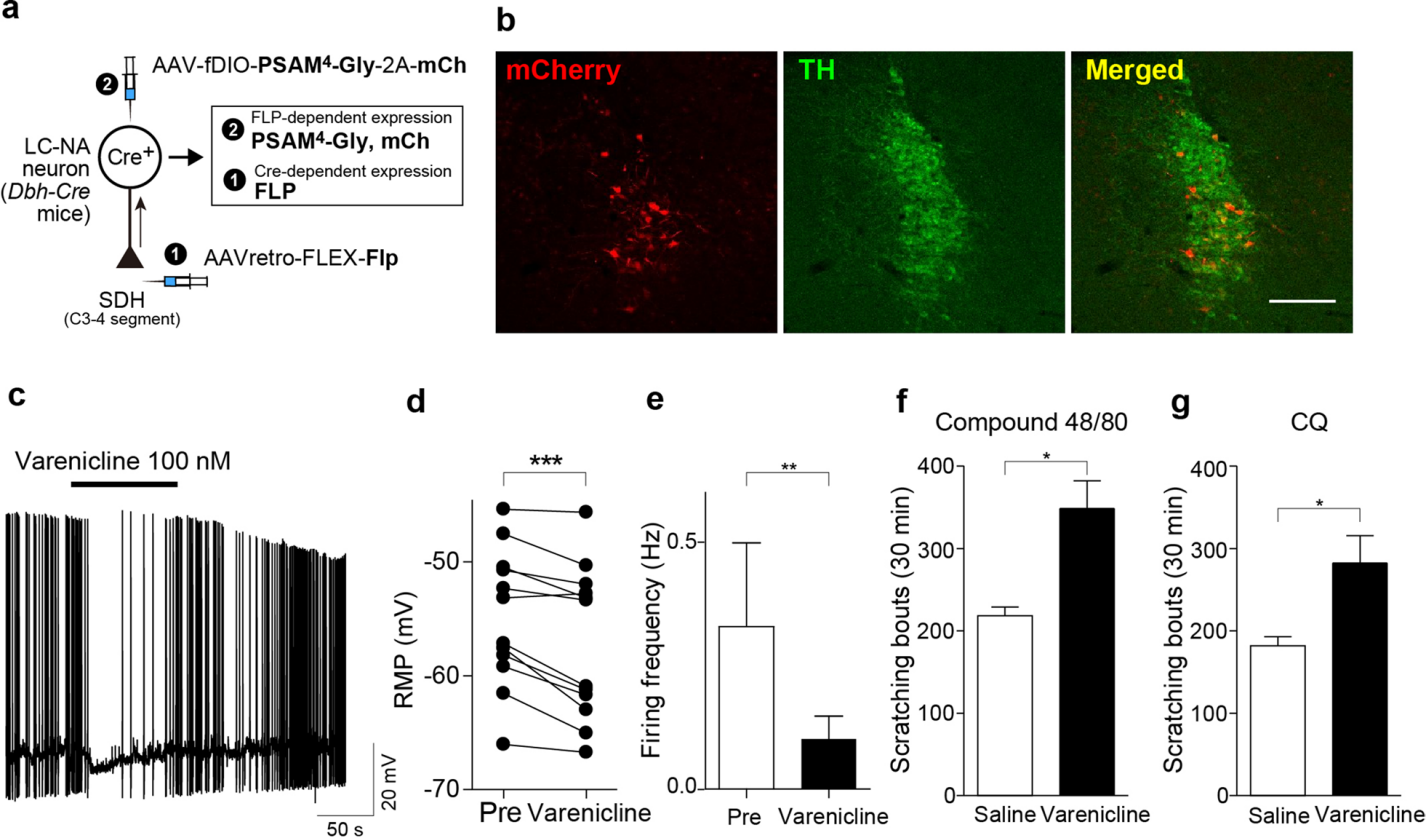
Chemogenetic silencing of descending LC-NAergic neurons enhances acute itch. **a** A schematic illustration of retrograde transduction strategy of PSAM^4^-GlyR expression in descending LC-NAergic neurons. **b** Expression of PSAM^4^-GlyR (detected by mCherry; red) in LC-NAergic neurons (visualized by TH immunostaining) in mice injected with the two AAV vectors. Scale bar, 200 μm. **c–e** Effect of varenicline (100 nM)-induced membrane hyperpolarization and spontaneous firing of mCherry^+^ neurons. The representative trace (**c**) and the summary data (**d**: RMP, n = 12, ***P < 0.001; **e**: spontaneous firing, n = 12, **P < 0.01). Scratching events induced by intradermal injection of compound 48/80 (50 μg/50 μl, **f**) or CQ (200 μg/50 μl, **g**) 20 min after varenicline (0.5 mg/kg) or saline administration (**f**: n = 5, **g**: n = 6, *P < 0.05). Data show the mean ± SEM

To silence the activity of descending LC-NAergic neurons, we expressed a new pharmacologically selective actuator module (PSAM) combined with chloride selective ion pore domain, PSAM^4^-GlyR channel [20], using AAV-fDIO-PSAM^4^-GlyR-2A-mCherry (Fig. 2a). PSAM^4^-GlyR expression (detected by mCherry) was also colocalized with TH immunofluorescence in the LC (Fig. 2b) (93.9 ± 1.7%: 304 TH^+^ neurons tested, n = 3 mice). Patch-clamp recording from mCherry^+^ LC neurons in hindbrain slices of these mice showed that bath application of varenicline (100 nM), a PSAM^4^-GlyR activator, hyperpolarized the resting membrane potentials and decreased the frequency of spontaneous firings of mCherry^+^ neurons (Fig. 2c–e). Using these mice, we found that varenicline administration increased scratching behaviors induced by both compound 48/80 (Fig. 2f) and CQ (Fig. 2g). Varenicline did not significantly increase the basal spontaneous scratching before injection of these pruritogens (saline, 4.6 ± 1.6, n = 10; varenicline, 9.1 ± 2.1, n = 11; P = 0.116, unpaired t test). These results suggested that SDH-projecting LC-NAergic neurons play a suppressive role in acute itch evoked by pruritogens injected into the skin.

### Facilitation of SDH NAergic signaling ameliorates chronic itch

**Fig. 3:**
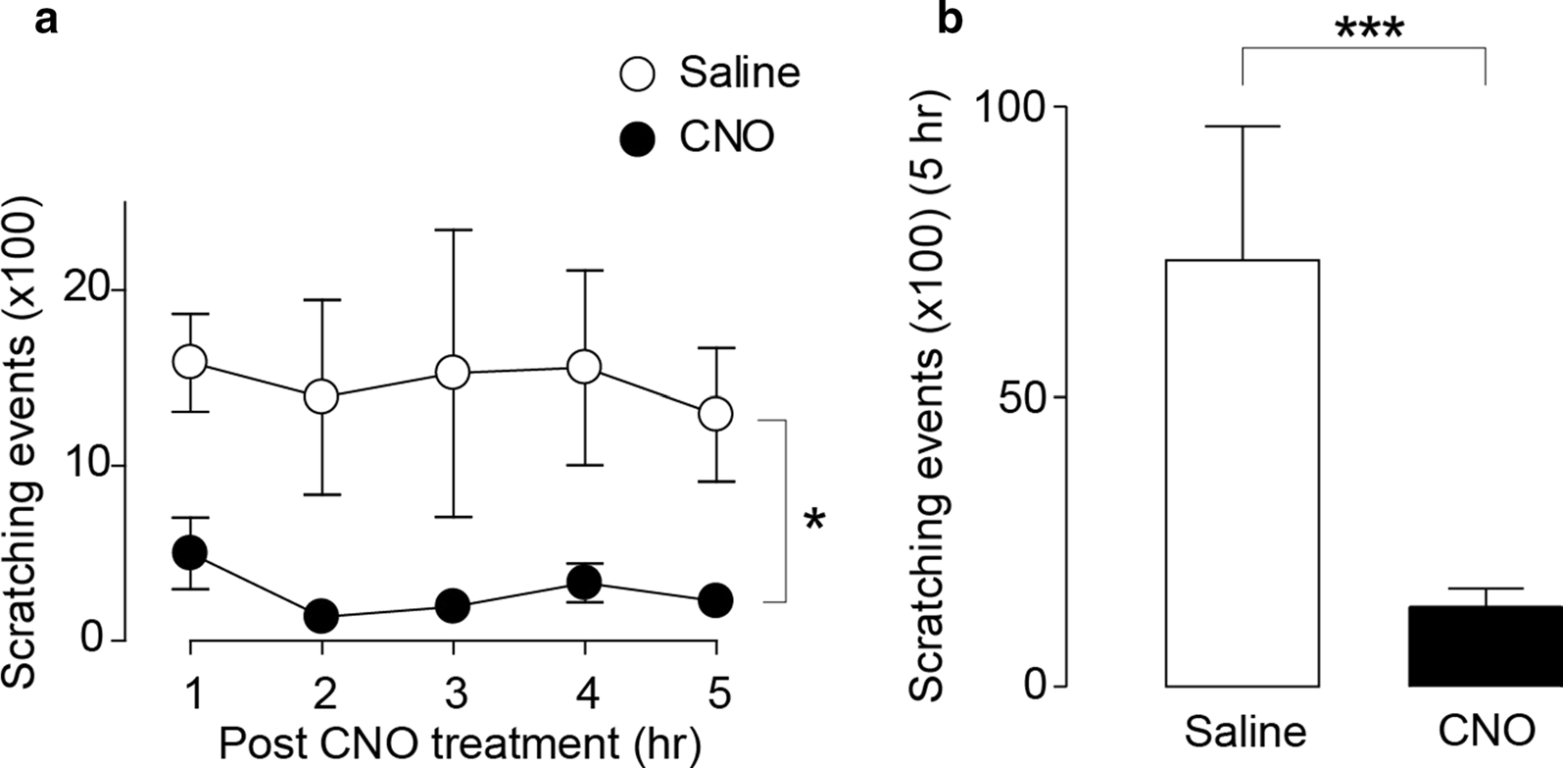
Chemogenetic activation of descending LC-NAergic signaling inhibits chronic itch. **a**, **b** Effect of CNO treatment on scratching behavior in DCP-treated mice that had expressed hM3Dq in the SDH-projecting LC-NAergic neurons by injection of two AAV vectors into the SDH and LC as shown in Fig. 1a. The time course of scratching behavior (**a**: n = 7, *P < 0.05) and the summary of total scratching behavior (**b**: n = 7, ***P < 0.001). Behavior was measured for 5 h after saline or CNO (10 mg/kg, i.p.) administration. Data show the mean ± SEM

**Fig. 4:**
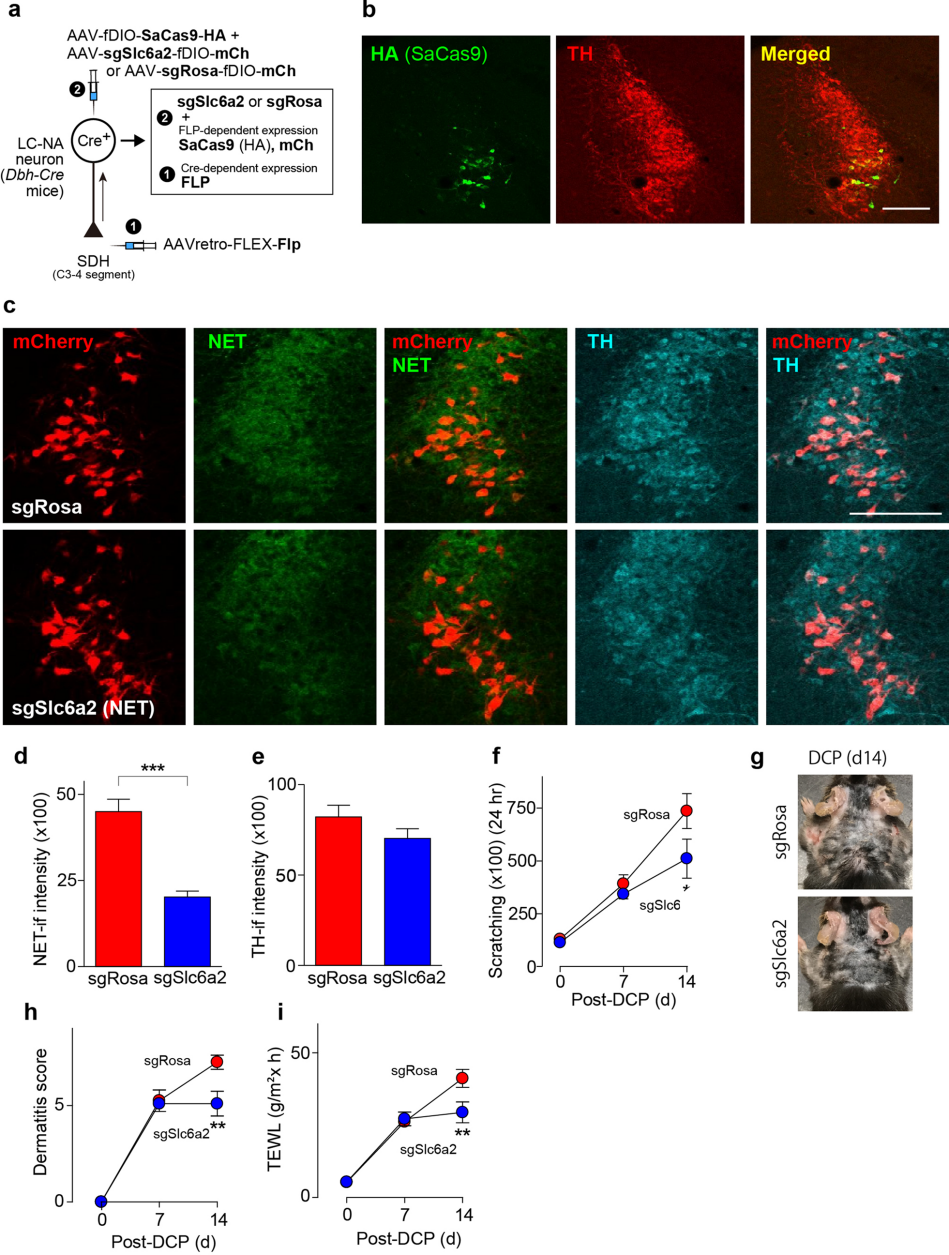
Descending LC-NAergic neuron-specific NA transporter knockout by CRISPR-Cas9 ameliorates chronic itch. **a** A schematic illustration of retrograde transduction strategy of SaCas9 and guide RNA [sgSlc6a2 for NA transporter (NET) and sgRosa (as control)] expression in descending LC-NAergic neurons. **b** Representative images of SaCs9 (detected by HA immunostaining, green) and TH (red) expression in LC-NAergic neurons. Scale bar, 200 μm. **c** Downregulation of NET (green) in descending LC-NAergic neurons (visualized by mCherry (red) and TH immunostaining (cyan)) in sgSlc6a2-expressing mice. Scale bar, 200 μm. Quantitative analysis of the immunofluorescence intensity of NET (**d**: n = 4 mice, ***P < 0.001) and TH (**e**: n = 4 mice). Effect of genome editing of NET on DCP-induced scratching behavior (**f**, *P < 0.05 vs. sgRosa-group on day 14), dermatitis [**g** (representative photomicrographs) and **h**, **P < 0.01 vs. sgRosa-group on day 14) and TEWL (**i**, **P < 0.01 vs. sgRosa-group on day 14) in DCP-treated mice (sgRosa, n = 8; sgSlc6a2, n = 10). Data show the mean ± SEM

The suppressive effect of descending LC-NAergic neurons was examined under a chronic itch condition using a model of contact dermatitis caused by applying diphenylcyclopropenone (DCP) to the skin [21, 22]. In DCP-treated mice in which hM3Dq was expressed in descending LC-NAergic neurons (Fig. 1a), scratching behavior was significantly reduced by CNO administration for 5 h (Fig. 3a, b). To determine the endogenous ability of descending LC-NAergic neurons to control chronic itch, we generated mice whose SDH-projecting LC-NAergic neurons lacked NA transporters (NET (encoded by *Slc6a2* gene), important for NA reuptake [23]) using a cell-type- and circuit-specific gene knockout using the CRISPR-Cas9 system [24]: AAVretro-FLEX-Flp was injected into the bilateral cervical SDH of *Dbh-Cre* mice, and later, an FLP-dependent *staphylococcus aureus* Cas9 (SaCas9)-expressing vector (AAV-fDIO-SaCas9-HA) and a single guide RNA (sgRNA)-expressing vector (AAV-sgSlc6a2-fDIO-mCherry or AAV-sgRosa-fDIO-mCherry (for control)) were injected into the bilateral LC (Fig. 4a). SaCas9 (detected by HA) and mCherry were mostly restricted to the ventral LC and were colocalized with TH (Fig. 4b, c) (sgRosa, 96.7 ± 0.5% (409 mCherry^+^ neurons tested, n = 4 mice); sgSlc6a2, 97.0 ± 0.9% (423 mCherry^+^ neurons tested, n = 4 mice)). A marked reduction of NET immunofluorescence in mCherry^+^ LC neurons was confirmed in mice with AAV-sgSlc6a2-fDIO-mCherry (Fig. 4c), and the intensity of NET immunofluorescence in mCherry^+^ LC-NAergic neurons was significantly reduced (Fig. 4d). The immunofluorescence intensity of TH in these neurons was indistinguishable between these two groups (Fig. 4e). In mice lacking NET in SDH-projecting LC-NAergic neurons, DCP-induced scratching behavior, dermatitis score and transepidermal water loss (TEWL, a dermatitis index) were significantly reduced on day 14 after the first DCP painting (Fig. 4f–i).

### NA facilitates inhibitory synaptic inputs on itch-transmission neurons via α_1A_-ARs

**Fig. 5:**
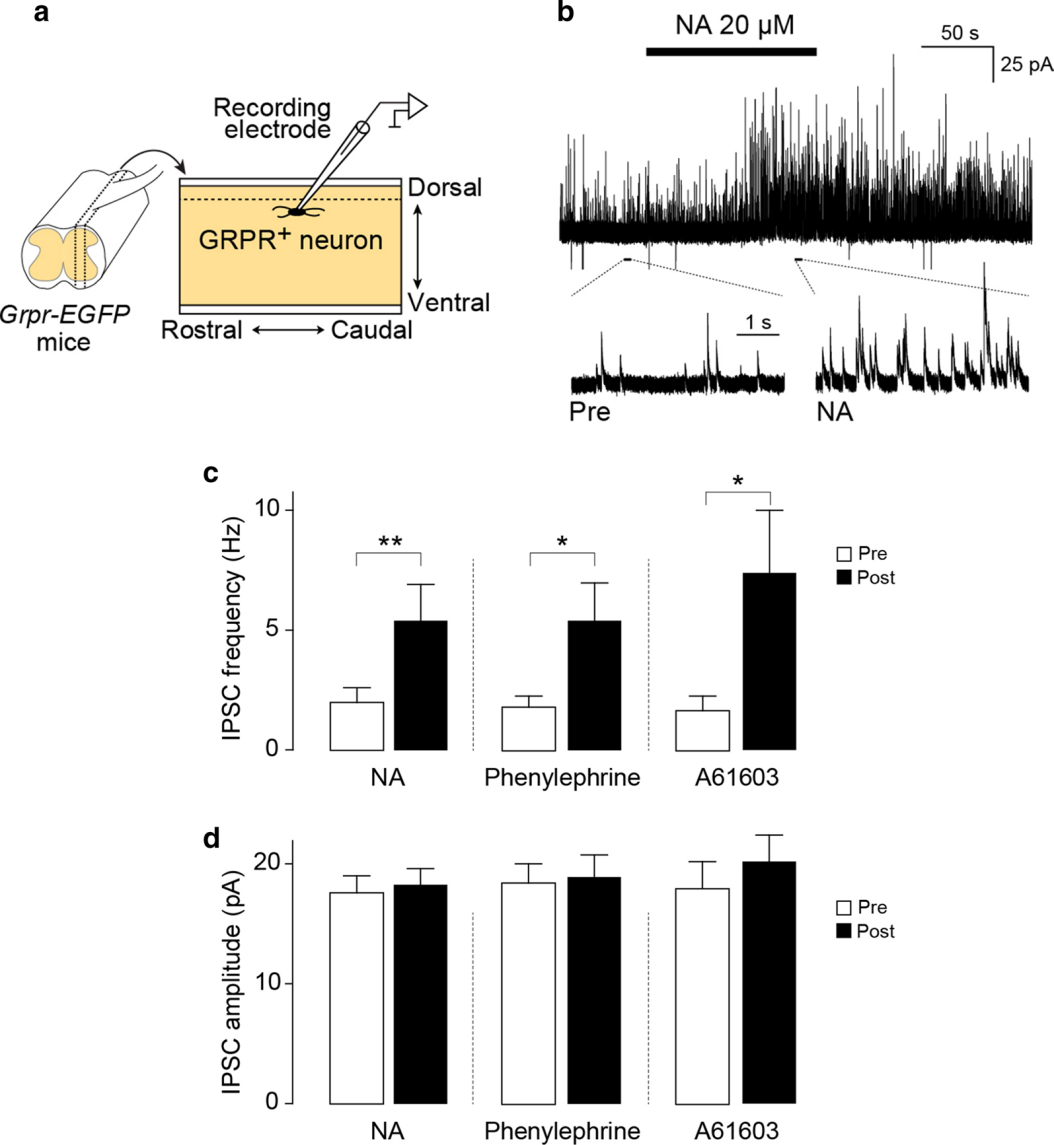
NA facilitates inhibitory synaptic inputs onto SDH GRPR^+^ neurons. **a** A schematic illustration of patch-clamp recording of GRPR^+^ neurons in cervical spinal cord slices (parasagittally cut) from *Grpr-EGFP* mice. **b** Representative trace of spontaneous IPSCs in SDH GRPR^+^ neurons before and after application of NA (20 μM). The lower panels show the traces in expanded time scale. Quantitative analysis of the effects of NA (20 μM, n = 13 cells), the α_1_-AR agonist phenylephrine (20 μM, n = 13 cells) and the α_1A_-AR agonist A61603 (5 μM, n = 6 cells) on the frequency (**c**) and amplitude (**d**) of spontaneous IPSCs in GRPR^+^ SDH neurons. *P < 0.05, and **P < 0.01. Data show the mean ± SEM

Behavioral data obtained in our study suggest that spinal NA would have an inhibitory effect on itch neurotransmission in the SDH. Our previous findings showed that activation of SDH inhibitory interneurons powerfully suppresses chronic itch [25], and GRPR^+^ neurons have been shown to be controlled by local inhibitory interneurons [26, 27]. Thus, we predicted that NA modulates inhibitory synaptic inputs onto GRPR^+^ neurons. To examine this, we performed patch-clamp recording from GRPR^+^ neurons using spinal cord slices from *Grpr-EGFP* mice [28, 29] and measured inhibitory postsynaptic currents (IPSCs) (Fig. 5a). Spontaneous IPSCs of GRPR^+^ neurons were markedly facilitated by the application of NA (Fig. 5b). The frequency of IPSCs was significantly increased by NA, while the average amplitude of IPSCs was not changed (Fig. 5d). A similar facilitation of IPSC frequency in GRPR^+^ neurons was observed after the application of the α_1_-AR agonist phenylephrine or the α_1A_-AR agonist A61603 [30] (Fig. 5c, d). These data suggest that spinal NA facilitates inhibitory synaptic inputs on GRPR^+^ neurons in the SDH presumably via α_1A_-ARs.

## Discussion

By using genetic approaches that enable cell-type and circuit-specific functional manipulation, we demonstrate for the first time that SDH-projecting LC-NAergic neurons powerfully control itch-related behavior. Indeed, stimulation of this pathway markedly suppressed scratching in models of histamine-dependent and -independent acute itch. Conversely, silencing the descending LC-NAergic pathway increased these itching behavioral responses. Thus, descending LC-NAergic neurons have an intrinsic ability to suppress acute itch. This hypothesis is supported by previous reports that intrathecally injected catecholaminergic neurotoxin 6-OHDA or α-AR antagonists increases scratching in acute itch models [10]. Furthermore, a marked suppression of scratching in mice with contact dermatitis was observed during acute stimulation of descending LC-NAergic neurons. This suggests that the inhibitory action of this pathway persists even under chronic itch conditions. The intrinsic potential of descending LC-NAergic neurons to suppress chronic itch was demonstrated by subsequent experiments, which showed that knocking out NET specifically in SDH-projecting LC-NAergic neurons using the CRISPR-Cas9 system resulted in the suppression of scratching in the DCP model. Consistent with our findings, NA and/or 5-HT reuptake inhibitors have also been shown to ameliorate chronic itch in mice [11] and in humans [12, 13]. Our findings, together with the findings of the aforementioned studies, suggest that the endogenous spinal NA released from descending LC-NAergic neurons may have an inhibitory effect on itch transmission in the SDH, although whether scratching behavior caused by other pruritogens is also modulated by descending LC-NAergic neurons is an important subject.

Given that intrathecal phenylephrine inhibits scratching [10], spinal α_1_-ARs could be responsible for the antipruritic effect of spinal NA. In the SDH, α_1_-ARs are preferentially expressed in inhibitory interneurons [31], and NA facilitates inhibitory synaptic inputs in the substantia gelatinosa [32]. GRPR^+^ SDH neurons have also been reported to form synaptic connections with inhibitory interneurons [26, 27]; however, whether NA facilitates inhibitory inputs to GRPR^+^ neurons was previously unknown. In our study, we demonstrated that NA facilitates inhibitory transmission in GRPR^+^ neurons. A similar facilitation was observed after α_1A_-AR agonist application, suggesting a role of α_1A_-ARs. The absence of change in the amplitude of IPSCs following NA release implies that NA acts on inhibitory interneurons that project onto GRPR^+^ neurons rather than acting directly on GRPR^+^ neurons. This is supported by recent studies that showed that the level of α_1A_-AR mRNA is high in galanin^+^ or preprodynorphin^+^ spinal interneurons [33], which are subsets of inhibitory interneurons that inhibit GRPR^+^ neurons [26, 27], but is low in GRPR^+^ neurons [31]. Thus, it is postulated that spinal NA derived from descending LC-NAergic neurons may activate these inhibitory interneurons via α_1A_-ARs, inhibit GRPR^+^ neurons, and suppress acute and chronic itch. However, from the data showing that inhibition of preprodynorphin^+^ inhibitory neurons by somatostatin causes spontaneous scratching [34] but chemogenetic inhibition of descending LC-NAergic neurons did not, it appears unlikely that LC-NAergic signals potently maintain basal activity of SDH inhibitory interneurons with a high level under normal conditions without any pruritic signals. Thus, LC-NAergic neurons could be activated in response to pruritic signals and produce their suppressive effect on scratching behavior.

In addition to α_1A_-ARs, α_2_-ARs could also play a role in regulating itch transmission. Indeed, α_2_-ARs are expressed in primary afferent pruriceptors that express either Mas-related G-protein coupled receptor member A3 or natriuretic peptides B [35], and also in GRPR^+^ SDH neurons [31]. Activating α_2_-ARs at presynaptic terminals of primary afferents reduces excitatory neurotransmission [36]. Intrathecal administration of α_2_-AR agonists inhibits acute itch responses [10, 37]. Therefore, the inhibitory effect of descending LC-NAergic neurons on acute and chronic itch could be the result of the combined effect of α_1A_- and α_2_-ARs signals. In addition, almost all neurons in the LC are known to produce NA [38], but subsets of LC neurons have also been shown to express some neuropeptides [39]. Additional experiments would be required to determine the role of neuropeptides in LC-NAergic neuron-derived modulation of GRPR^+^ SDH neurons and itch behavior in the future.

LC-NAergic neurons also send ascending projections throughout the brain that modulate several key brain functions [38, 40]. Recently, it was reported that the activation of LC-NAergic neurons that project to the anterior cingulate cortex induces scratching behaviors [41]. Thus, it is possible that ascending and descending LC-NAergic neurons play a distinct role in the control of itch behavior. However, how their neuronal activity is differentially regulated under acute and chronic itch conditions remains unclear.

In summary, we showed that SDH-projecting LC-NAergic neurons have an intrinsic potential to suppress acute and chronic itch and that NA facilitates inhibitory synaptic inputs onto GRPR^+^ SDH neurons presumably via α_1A_-ARs. Our finding that descending LC-NAergic neuron-specific NET knockout exerts an ameliorating effect on chronic itch and previous data showing that NA and/or 5-HT reuptake inhibitors reduce chronic itch in mouse models [42], including the DCP model [11], and in humans [12, 13], indicate that descending LC-NAergic signaling could be targeted to treat chronic itch.

## Methods

### Animals

C57BL/6J mice (Jackson Laboratory, USA), *Grpr-EGFP* mice (STOCK Tg(*Grpr-EGFP*)^PZ62Gsat/Mmucd^) (MMRRC, USA) [28] and *Dbh-Cre* mice (B6.Cg-Tg(B6.Cg-Tg(*DBH-cre*)^9-9Koba/KobaRbrc^) (RIKEN BRC, Japan) [19] were used. All mice used were male and 8–12 weeks of age at the start of each experiment and were housed at 22 ± 1 °C with a 12-h light–dark cycle. All animals were fed food and water ad libitum. All animal experiments were conducted according to relevant national and international guidelines contained in the ‘Act on Welfare and Management of Animals’ (Ministry of Environment of Japan) and ‘Regulation of Laboratory Animals’ (Kyushu University) and under the protocols approved by the Institutional Animal Care and Use committee review panels at Kyushu University.

### Immunohistochemistry

Immunohistochemical experiments were performed according to the methods in our previous study [22]. Mice were deeply anesthetized by i.p. injection of pentobarbital and perfused transcardially with phosphate buffered saline (PBS), followed by ice-cold 4% paraformaldehyde (PFA)/PBS. The brains were removed, postfixed in the same fixative for overnight at 4 °C and placed in 30% sucrose solution for two overnight at 4 °C. Transverse brain sections (40 μm) were made and immunostained. Primary and secondary antibodies used were listed below. Primary antibodies: polyclonal rabbit anti-TH (1:1000, Merck Millipore, A152, Germany), polyclonal sheep anti-TH (1:1000, Merck Millipore, A1542), monoclonal guinea pig anti-NET (1:2000, Frontier Institute, AB_2571810, Japan), monoclonal rabbit anti-HA-Tag (1:1000, Cell Signaling, 3724, USA), monoclonal rat anti-mCherry (1:2000, Thermo Fisher Scientific, M11217, USA), monoclonal rabbit anti-c-FOS (1:1000, Cell Signaling, 2250), and secondary antibodies: Alexa Fluor 488 and/or 546 and/or 405 (1:1000, Molecular Probes). Immunofluorescence images were obtained with a confocal laser microscope (LSM700, Carl Zeiss, Germany). Fluorescent intensity of TH or NET was quantified using Fiji (https://fiji.sc).

### Vector construction and AAV production

The methods were in accordance with our previous studies [29, 43]. The gene encoding FLP (Addgene #55637) [44] and PSAM^4^-Gly (Addgene #119739) [20] were subcloned into pENTER plasmids (Thermo Fisher Scientific, MA, USA). AAV vector plasmids of FLP-dependent gene transduction were generated from pAAV-EF1a-fDIO-EYFP (Addgene #55641) [44], by substituting the EYFP with PSAM^4^-Gly-2A-mCherry, HA-hM3Dq-2A-mCherry [17], mCherry and SaCas9-HA [45]. To reduce the packaging size of FLP-induced SaCas9-HA expression vector, we generated pZac2.1-CMVmini-fDIO-SaCas9-HA from pZac2.1-CMVmini-SaCas9-HA vector (Addgene #78601) [45], by substituting the SaCas9 with fDIO-SaCas9-HA. Synthetic oligonucleotides including targeting sequence for exon3 of Slc6a2 (5′-AATACAAGTTCACACCAGCTG-3′) or for Rosa locus (5′-CTCTAGAGTCGCAGATCCTC-3′) with the targeting site in the original pENTER-U6-sgBsa1 plasmid [29]. The each resulting U6-sgRNA cassette was transferred into pAAV-EF1a-fDIO-mCherry plasmid. rAAV2-retro helper plasmid was purchased from Addgene (#81070) [18]. The rAAV vectors were produced from human embryonic kidney 293 (HEK293) cells with triple transfection (each pZac2.1 or pAAV plasmid; pAAV2/9 or rAAV2-retro trans plasmid; pAd DeltaF6, adenoviral helper plasmid). Viral lysate was harvested at 72 h post-transfection and lysed by freeze-and-thaw cycles, purified through two rounds of CsCl ultracentrifugation, and then concentrated using Vivaspin 20 ultrafiltration units (SARSTEDT, Germany). The genomic titer of rAAV was determined by Pico Green fluorometric reagent (Molecular Probes, USA) following denaturation of the AAV particle. Vectors were stored in aliquots at − 80 °C until use [43].

### Intra-SDH and intra-LC injection of rAAV vector

Intra-SDH injection was in accordance with our previous studies [29, 43]. Mice were deeply anesthetized by subcutaneous injection of ketamine (100 mg kg^−1^) and xylazine (10 mg kg^−1^). Mice was shaved on the back of the neck, and the skin was incised at C3–C5. The muscle on C3–C5 vertebrae was opened with a retractor, and mice were attached with a head-holding device (SR-AR, NARISHIGE, Japan). Paraspinal muscles around the left side of the interspace between C3 and C4 vertebrae were removed, and the dura mater and the arachnoid membrane were carefully incised using the tip of a 30G needle to make a small window to allow a glass microcapillary insert directly into the SDH. The glass microcapillary was inserted into the SDH (150–200 μm in depth from the surface of the dorsal root entry zone) through the small window (approximately 500 μm lateral from the midline). rAAV solution was pressure-ejected (100 nL min^−1^) for 5 min (approximately 500 nL) using the Micro Syringe Pumps (SYS-micro4, WPI, USA) bilaterally. After microinjection, the inserted glass microcapillary was removed from the SDH, the skin was sutured with 3–0 silk, and mice were kept on a heating light until recovery. For intra-LC injection, rAAV solutions were bilaterally injected (approximately 300 nl in one site) adjacent to the LC [rostrocaudal (RC), 5.6 mm; mediolateral (ML), ± 1 mm; dorsoventral (DV) 3.7 mm]. We used virus-injected mice for further analysis 21 days or more after the last injection of AAV vectors. The used viral titers were as follows: AAVretro-EF1α-FLEX-Flp, 5 × 10^12^ genome copies (GC) ml^−1^; AAV2/9-EF1α-fDIO-PSAM^4^-Gly-2A-mCherry or HA-hM3Dq-2A-mCherry, 5.0 × 10^12^ GC ml^−1^; AAV2/9-CMVmini-fDIO-SaCas9-HA, 5.0 × 10^12^ GC ml^−1^; AAV2/9-U6-sgSlc6a2-EF1α-fDIO-mCherry or AAV2/9-U6-sgRosa-EF1α-fDIO-mCherry, 2.0 × 10^12^ GC ml^−1^.

### Electrophysiology

According to our previous study [29], mice were deeply anesthetized with ketamine (100 mg kg^−1^) and xylazine (10 mg kg^−1^) and the cervical spinal cord (SC) or the brain were removed and placed into a cold high sucrose artificial cerebrospinal fluid (sucrose aCSF) (250 mM sucrose, 2.5 mM KCl, 2 mM CaCl_2_, 2 mM MgCl_2_, 1.2 mM NaH_2_PO_4_, 25 mM NaHCO_3_ and 11 mM glucose). A parasagittal SC slice (250–300 μm thick) or colonal hindbrain slices (300 μm thick) were made using a vibrating microtome (VT1200, Leica, Germany) and then the slices kept in oxygenated artificial cerebrospinal fluid (aCSF) solution (125 mM NaCl, 2.5 mM KCl, 2 mM CaCl_2_, 1 mM MgCl_2_, 1.25 mM NaH_2_PO_4_, 26 mM NaHCO_3_ and 20 mM glucose) at room temperature (22–25 °C) for at least 30 min. The SC or brain slice was then put into a recording chamber where it was continuously superfused with aCSF solution at 25–28 °C at a flow rate of 4–6 mL min^−1^. We used two kinds of internal solution, K-gluconate solution (125 mM K-gluconate, 10 mM KCl, 0.5 mM EGTA, 10 mM HEPES, 4 mM MgATP, 0.3 mM NaGTP, 10 mM phosphocreatine, pH 7.28 adjusted with KOH) for membrane potential recording, or Cs-based solution (120 mM CsMeSO_4_, 15 mM CsCl, 10 mM HEPES, 5 mM QX-314, 4 mM MgATP, 0.3 mM Na_2_GTP, 0.2 mM EGTA, 10 mM TEA-Cl, 8 mM NaCl, pH 7.28 adjusted with CsOH) for IPSC recording. Whole-cell patch-clamp recordings were made from fluorescent-labeled neurons. Recordings were made using Axopatch 700B amplifier and pCLAMP 10.4 acquisition software (Molecular Devices, USA). The data were digitized with an analog-to-digital converter (Digidata 1550, Molecular Devices), stored on a personal computer using a data acquisition program (ClampeX version 10.4, Molecular Devices) and analyzed using a software package (Clampfit version10.4, Molecular Devices) or minianalysis (Synapsoft Inc, USA). Membrane potentials were recorded in a current-clamp mode. IPSCs were recorded in the voltage-clamp mode at a holding potential of 0 mV. All drugs were dissolved into the aCSF solution and bath-applied for 2 min. Drugs used were varenicline tartrate (100 nM, Tocris Bioscience, UK), l-noradrenaline hydrochloride (20 µM, Sigma, USA), (R)-(-)-phenylephrine hydrochloride (20 µM, Wako, Japan) and A61603 (Tocris Bioscience).

### Mouse models of acute and chronic itch

Behavioral tests using models of acute and chronic itch was performed by the methods in our previous studies [22, 29]. For acute itch models, mice were shaved on the back until one day before injection. CNO (10 mg kg^−1^, Enzo Life Science, USA) or varenicline (0.5 mg kg^−1^; Tocris Bioscience) were dissolved in saline and intraperitoneally administered 20 min before intradermal injection of pruritogens [chloroquine (200 µg/50 µl; C6628, Sigma) and compound 48/80 (50 µg/50 µl; C2313, Sigma)] into the shaved back. After the injection, the mouse was placed in a plastic chamber (11 cm in diameter, 10 cm high). Hind limb scratching behavior directed toward the injection site was observed for 30 min. One scratch was defined as a lifting of the hind limb toward the injection site and then placing the limb back on the floor, regardless of how many scratching strokes took place between those two movements [6]. For the basal spontaneous scratching behavior analysis, we counted the scratching behaviors for 15 min after intraperitoneally administration of CNO, varenicline or saline. For a chronic itch model of contact dermatitis, mice were shaved on the back and topically applied by painting 0.2 ml of 1% diphenylcyclopropenone (DCP; Wako) dissolved in acetone under isoflurane anesthesia. Seven days after the first painting (day 7), DCP was painted again on the same area of skin. Seven days later (day 14), measurement of scratching behavior and other experiments [immunohistochemistry or transepidermal water loss (TEWL: see below)] were performed. Scratching behavior in mice was automatically detected and objectively evaluated using MicroAct (Neuroscience, Japan) in accordance with a method described previously [22]. Under isoflurane anesthesia, a small Teflon-coated magnet (1 mm in diameter, 3 mm in length, Neuroscience) was implanted subcutaneously into the hindpaws of the mice at least 1 day before the first recording. Each mouse with implanted magnet was placed in an observation chamber (11 cm in diameter, 18 cm high) with food and tap water, surrounded by a round coil. Movement of magnets implanted subcutaneously into the hindpaws induced electric currents in the coil, which were amplified and recorded by MicroAct software. The analysis parameters for detecting scratch movements were as follows: threshold, 0.07 V; event gap, 0.2 s; minimum duration, 0.2 s; maximum frequency, 35 Hz; minimum frequency, 2 Hz; minimum beats, 2. Scratching behavior was shown as the number of total scratching responses over 5 or 24 h.

### Measurement of transepidermal water loss

Transepidermal water loss (TEWL) was measured using the Tewameter TM300 system and a multi-probe adaptor (CK electronic, Germany), in accordance with manufacturer instructions and our previous studies [29]. Under isoflurane anesthesia, the probe collar was placed on the surface of the skin on the animal’s back for 20–30 s. Measurements were obtained twice for the left and right sides of the skin, and the values were averaged.

### Evaluation of dermatitis

Severity of dermatitis of the face, ears, and the rostral part of the body was assessed as previously described [22, 46]: no symptoms (score 0), mild (score 1), moderate (score 2), and severe (score 3). This scoring system was separately applied to the severity of erythema/hemorrhage, edema, excoriation/erosion, and scaling/dryness. The total score (minimum 0, maximum 12) was expressed as the sum of each score of the above four symptoms.

### Statistical analysis

All data are shown as the mean ± SEM. Statistical significance of differences was determined using paired t test (Fig. 2d, 5c, d) unpaired t test (Fig. 1d, e, 4d, e), unpaired t test with Welch's correction (Fig. 2f, g, 3b), Wilcoxon signed rank test (Fig. 2e), two-way repeated measures ANOVA with post hoc Bonferroni test (Fig. 3a, 4f, h, i) using GraphPad Prism 4 and 7 software. Differences were considered significant at P < 0.05.

## Data Availability

All data needed to evaluate the conclusions in the paper are present in the paper.

## Abbreviations

AAV: Adeno-associated virus
AR: Adrenaline receptor
CNO: Clozapine-N-oxide
CQ: Chloroquine
CRISPR: Clustered regularly interspersed short palindromic repeat
DBH: Dopamine-β-hydroxylase
DCP: Diphenylcyclopropenone
EGFP: Enhanced green fluorescent protein
FACS: Fluorescent activated cell-sorting
FLP: Flippase
GRP: Gastrin-releasing peptide
GRPR: Gastrin-releasing peptide receptor
hM3Dq: Modified human muscarinic Gq-protein-coupled receptor
IPSC: Inhibitory postsynaptic current
LC: Locus coeruleus
NA: Noradrenaline
NET: Noradrenaline transporter
PSAM: Pharmacologically selective actuator module
SDH: Spinal dorsal horn
SaCas9: *Staphylococcus aureus* Cas9
TEWL: Transepidermal water loss
TH: Tyrosine hydroxylase
